# Feasibility and efficiency of microalgae cultivation for nutrient recycling and energy recovery from food waste filtrate

**DOI:** 10.1371/journal.pone.0315801

**Published:** 2025-02-05

**Authors:** Yanghang Chen, Wing-Wai Wan, Kai-Hui Cui, Bonnie Pui-Ying Lau, Fred Wang-Fat Lee, Steven Jing-Liang Xu

**Affiliations:** 1 Laboratory of Marine Biodiversity, Third Institute of Oceanography, Ministry of Natural Resources, Xiamen, China; 2 Department of Applied Science, School of Science and Technology, Hong Kong Metropolitan University, Ho Man Tin, Kowloon, Hong Kong; 3 State Key Laboratory of Marine Pollution and Department of Chemistry, City University of Hong Kong, Kowloon, Hong Kong; Luleå University of Technology, SWEDEN

## Abstract

With the continuous growth of economic and population, the generation of food waste has significantly increased in recent years. The disposition of food waste, typically through incineration or landfill, can lead to severe health and environmental problems, accompanied by high additional costs. However, the leachate produced from food waste during collection, transportation and landfill operations predominantly contains high levels of nutrients necessary for microalgae growth. The integration of microalgae cultivation into waste treatment for nutrient recycling presents a potential route for energy recovery from food waste. Therefore, this study was conducted to evaluate the feasibility of microalgae cultivation for food waste filtrate treatment. In addition, the optimal cultivation conditions and nutrient removal efficiency for microalgae in food waste filtrate treatment were investigated. The results indicated that *Cyanobacterium aponinum* exhibited the highest growth rate (0.530 cells d^-1^) and maximum cell density (9.6 × 10^6^ cells mL^-1^) among eight potential microalgal species in 10% food waste filtrate treatment under 10,000 lux and 32°C. It was also observed that *C*. *aponinum* had significantly higher biomass productivity and nutrient removal efficiency under a 5% CO_2_ concentration. The successful cultivation of *C*. *aponinum* demonstrated that food waste filtrate could be a promising growth medium, reducing the high cost of cultivation with synthetic medium. However, further efforts should be made to utilize microalgae in food waster filtrate treatment, transitioning from laboratory condition to a pilot scale.

## Introduction

Globally, approximately one-third (1.3 billion tons) of the edible food initially produced for human consumption is wasted annually, a phenomenon referred to as food waste [1]. With escalating economic and population growth, the generation of food waste has significantly increased in recent years, particularly in the Asian region [2]. In Hong Kong, the volume of disposed food waste from commercial and industrial sectors rose approximately 2.5 times from 2002 to 2013 [3]. The impact of food waste is considerable, encompassing the vast amounts of fertile land wasted in food production and the contribution to greenhouse gas emissions via carbon footprint [1, 2]. Presently, the most prevalent methods of food waste disposal are incineration or landfill, both of which can lead to severe health and environmental issues [4]. Incineration of food waste results in the release of certain persistent organic pollutants, while leachate from landfills can cause groundwater pollution and, consequently, contamination of nearby waterways [2, 5]. Furthermore, the incineration of food waste, which consists of over 80% moisture content, and the treatment of leachate by specific installations are highly energy-intensive and incur additional costs, respectively [6]. However, leachate from food waste provides an excellent medium for microalgal growth by supplying essential nutrients such as nitrogen and phosphate. Compared to traditional media, which account for a large portion of microalgae cultivation costs, food waste filtrate can reduce expenditure [7]. Consequently, microalgae-based food waste filtrate treatment is currently garnering increasing attention [8]. The most commonly used species for food waste filtrate treatment is the green algae *Chlorella pyrenoidosa*, which has demonstrated removal efficiencies exceeding 90% for total nitrogen (TN) and total phosphorus (TP), and approximately 70% for chemical oxygen demand (COD) [9, 10].

On the other hand, deriving biodiesel from microalgae presents a potential solution to the energy crisis. As traditional fossil fuel is a finite energy source and a primary contributor to global warming, research into sustainable biofuels has been strongly encouraged by professionals in recent decades [11]. Microalgae have garnered increasing attention as one of the most promising renewable feedstocks for biodiesel production, owning to their high growth rates and lipid contents [12]. Through photosynthesis, lipids accumulate within microalgae, and approximately 3%-8% of solar energy can be converted into biomass, compared to a mere 0.5% photosynthetic yield from terrestrial plants [13]. However, numerous challenges persist, such as the high cost of microalgae cultivation, which is considered a primary factor inhibiting the development of large-scale production [14]. To date, only a few industrial facilities have produced biodiesel from microalgae [11]. The use of wastewater, including food waste filtrate, as a nutrient source for microalgae cultivation is deemed an economically feasible and environmentally friendly approach to enhance the efficiency and sustainability of microalgal biofuel systems [15]. The integration of food waste filtrate treatment into microalgae cultivation is highly advantageous, not only for the cost-effectiveness of microalgal biomass yield for biodiesel production, but also for the cost savings associated with nutrient removal [16].

Currently, numerous advanced countries, including America, Mexico, and Australia, are showing significant interest in developing the bio-technique of wastewater treatment with microalgae [17]. However, while many researchers have dedicated their efforts to treating industrial or municipal wastewater with microalgae, there is limited information or results on food waste filtrate treatment by microalgae. This is primarily due to the different filtrate composition of food waste compared to common wastewater. The oil and grease content in food waste filtrate is much higher than that in industrial or municipal wastewater, which can inhibit treatment efficiency. Consequently, not all microalgae strains can be applied in food waste filtrate treatment [18, 19]. Based on findings and experiences from previous research, high growth performance, lipid productivity, and nutrient removal ability are the criteria for selecting microalgae for food waste filtrate treatment. These may be influenced by certain factors: (1) Light intensity affects the photosynthetic rate and desired product (such as lipid) accumulation of microalgae. Optimal light intensity varies significantly among different microalgae strains, with ranges for microalgal growth, lipid production, wastewater remediation, and other applications are from 20 to 2000 μmol m^-2^ s^-1^ [20, 21]. Considering cultivation cost and energy consumption, the optimal light intensity should be chosen to achieve the most efficient level of cell growth and desired product [22, 23]. (2) Temperature, one of the most distinctive environmental factors regulating reproduction of microalgae, also influences cell size, biochemical composition, and nutrient requirements. Depending on the strain, region and season, microalgae can grow under a broad range of temperatures from 15°C to 40°C [24, 25]. Additionally, temperature may be a key factor affecting photoinhibition by impacting the microalgal growth rate [26]. (3) Carbon dioxide (CO_2_), the primary carbon source for photoautotrophic microalgae, is essential for photosynthesis and, consequently, the growth and reproduction of microalgae [27]. The carbon fixation of microalgae is mostly used for respiration, serving as an energy source or raw material in extracellular formation. The microalgal growth rate will be directly related to the supply of carbon fixation rate [28].

In the current study, eight potential microalgal species were cultivated in varying concentrations of food waste filtrate. Following the identification of capable microalgae for food waste filtrate treatment through comparison of cell density and growth rate, the optimal cultivation conditions, including light intensity, temperature and CO_2_ concentration, as well as nutrient removal efficiency under different CO_2_ concentrations, were also investigated. Our aim was to evaluate the feasibility of microalgae cultivation for food waste filtrate treatment and the potential for industrial production of microalgae biomass from food waste filtrate.

## Materials and methods

### Food waste filtrate

The filtrate from food waste was obtained from the restaurants located in the large shopping mall Olympic City II in Hong Kong, China. As part of a cooperative scheme, all the operation was permitted by the management of Olympic City II. The food waste was gathered in a plastic container and subsequently filtered using a 1 cm filter to eliminate solid food waste. The collected filtrate was then transferred into a 20 L plastic bottle and stored at 4°C.

### Microalgae strains and culture

Eight strains of microalgae, provided by the Algal Library of Hong Kong Metropolitan University (HKMU), were maintained in L1 medium [29] with 0.22 μm-filtered seawater with a salinity of 30 ± 1 ‰ (Table 1). The natural seawater was sourced from Tolo Harbour, Hong Kong, China, and was autoclaved prior to use. The cells were cultured at a temperature 24 ± 1°C and a light intensity of 6,000 lux, using cool white light under a 12 h: 12 h light-dark cycle. Microalgae in the exponential growth phase were utilized for the experiments.

**Table 1:** Microalgae strains for cultivation experiment.

Strain number	Species	Source
H2	*Cyanobacterium aponinum*	Wastewater plant, HK
STK-2	*Dunaliella tertiolecta*	Sha Tau Kok, HK
904	*Tetraselmis suecica*	Coastal water, Europe
Affine	*Alexandrium affine*	Junk Bay, HK
HK5	*Prorocentrum minimum*	Junk Bay, HK
AD1	*Prorocentrum triestinum*	Yim Tin Tsai, HK
3DS	*Scrippsiella trochoidea*	Coastal water, China
WWS	undefined	Wastewater plant, HK

### Experimental setup

#### Cultivation of multiple microalgal strains in food waste filtrate

The food waste filtrate was diluted to various concentrations (10%, 30%, 80%, and 100%) using sterilized seawater with a salinity of 30 ± 1 ‰ (the same source as used for the L1 medium). Eight selected strains of microalgae were cultivated in these different concentrations of food waste filtrate, each in a 250 mL flask. The total volume for each experiment was 100 mL, with cells cultivated in 100% sterilized seawater serving as controls. Both the control and experimental groups were cultured in triplicate under the same conditions as those described in the section “Microalgae strains and culture”. A natural air current of 0.5 L min^-1^ was provided at the base of the flask. Algal samples were collected daily and fixed with Lugol’s solution for cell growth determination over a 14-day period.

#### Optimum growth conditions of *C*. *aponinum*

Given that *C*. *aponinum* was identified as a potential microalga for food waste filtrate treatment in the section “Cultivation of multiple microalgal strains in food waste filtrate”, three sets of experiments were conducted to investigate its optimal growth conditions.

*a) Light intensity*. *C*. *aponinum*, with an initial cell density of 5.0 × 10^5^ cells mL^-1^, was cultivated under various light intensities (6,000, 8,000, 10,000, 12,000, and 14,000 lux). All groups were cultured in triplicate under the same conditions as those described in the section “Microalgae strains and culture”. A natural air current of 0.5 L min^-1^ was provided at the base of the flask. Algal samples were collected daily and fixed with Lugol’s solution for cell growth determination over a 14-day period.

*b) Temperature*. *C*. *aponinum* at an initial cell density of 5.0 × 10^5^ cells mL^-1^ was cultivated under five temperature ranges (24°C, 28°C, 32°C, 36°C, and 40°C). All the groups were cultured in triplicate under the same conditions as those described in the section “Microalgae strains and culture”. A natural air current with 0.5 L min^-1^ was provided at the flask bottom. The algal samples were collected and fixed with Lugol’s solution daily for cell growth determination over a 14-day period.

*c) CO*_*2*_ *concentration*. *C*. *aponinum*, with an initial cell density of 5.0 × 10^5^ cells mL^-1^, was cultivated under the same conditions as those described in the section “Microalgae strains and culture”, with a natural air current of 0.5 L min^-1^ provided at the base of the flask. In addition to the normal air current, various concentrations (2.5%, 5%, 7.5%, and 10%) of CO_2_ were also supplied. All groups were cultured in triplicate. Algal samples were collected daily and fixed with Lugol’s solution for cell growth determination over a 14-day period.

#### Efficiency of nutrient removal by *C*. *aponinum* under different CO_2_ concentrations

*C*. *aponinum*, in the exponential growth phase, was inoculated into the food waste filtrate at a 10% concentration, which was diluted with sterilized seawater with a salinity of 30 ± 1 ‰. Based on the results of optimal growth conditions detailed in the section “Optimum growth conditions of *C*. *aponinum*”, cells were cultured at a temperature of 32 ± 1°C and a light intensity of 10,000 lux, using cool white light under a 12 h: 12 h light-dark cycle. A natural air current, along with 5% and 10% concentrations of CO_2_ at a rate of 0.5 L min^-1^, were provided at the base of the flask, respectively. All groups were cultured in triplicate. Initial and final samples were collected on day 0 and day 14, respectively, for nutrient analysis.

### Sample analysis

#### Cell growth

Algal growth was determined by cell counting under a light microscope using a Sedgwick-Rafter Cell Counter. The specific growth rate (x) was calculated using the following equation: x = (ln N_1_—ln N_0_) / (t1—t_0_), where N_0_ and N_1_ represent the cell density at time t_0_ and t_1_, respectively.

#### Nutrients analysis

A 50 mL volume of culture was collected to determine nutrient concentrations. Initial and final samples were filtered through a 1 μm filter (Whatman, USA). The filtrates were then appropriately diluted and analyzed for Total Nitrogen (TN), Total Phosphorus (TP), ammonia, nitrate, phosphate, and Chemical Oxygen Demand (COD) following the Hach DR 2800 Portable Spectrophotometer Manual. Nutrient removal efficiency (R_x_) was calculated from the following equation: R_x_% = (C_x0_ –C_x1_) / C_x0_ × 100%, where C_x0_ and C_x1_ represent the initial and final concentrations on day 0 and day 14, respectively.

### Statistical analysis

All values were expressed as the mean ± standard deviation (SD). Student’s *t* test and one-way analysis of variance (ANOVA) followed by Tukey test were used to analyze differences between the experimental and control groups (or between different culture conditions). A value of *p* < 0.05 was considered statistically significant. All statistical analyses were performed using the software package SPSS 22.0.

## Results and discussion

### Growth of multiple potential microalgae cultured in food waste filtrate

**Fig 1:**
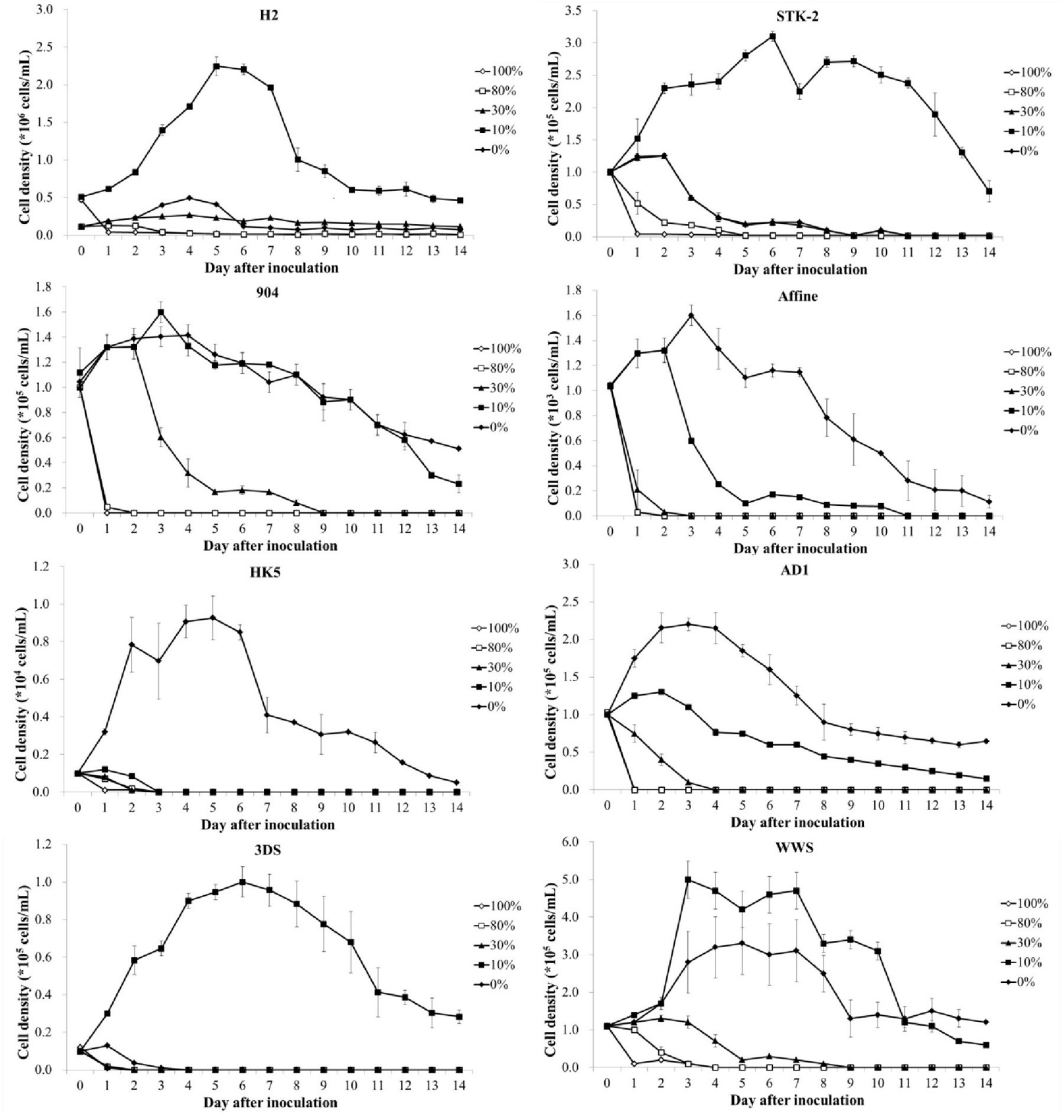
Growth of eight selected algal strains cultured in various concentrations of food waste filtrate. The mean and standard deviation of three replicates are shown.

To investigate the potential for bioremediation of food waste filtrate using microalgae, eight selected algal stains were cultivated in various concentrations of food waste filtrate. Fig 1 shows the growth performance of all these strains when cultured in different food waste filtrate concentrations. A comparison of cell densities between day 0 and day 6 for each treatment (Table 2) revealed that none of the selected algal species survived beyond 6 days when cultured in high concentrations of food waste filtrate. When cultivated in 30% food waste filtrate, only strain H2 exhibited a 228% increase in cell density. Under 10% food waste filtrate treatment, the cell densities of strains WWS, H2, and STK-2 increased by 800%, 383%, and 300%, respectively. These results suggest that the optimal concentration for microalgae treatment of food waste filtrate is 10%, and that microalgae struggle to grow in high concentrations (≥ 30%) of food waste filtrate.

**Table 2:** Comparison of cell densities of eight selected algal strains under various concentrations of food waste filtrate treatment between day 0 and day 6. “Death” indicates that no cells could be counted in samples.

	100% filtrate	80% filtrate	30% filtrate	10% filtrate	0% filtrate (Control)
H2	< 1%	16%	228%	383%	123%
STK-2	Death	< 1%	15%	300%	16%
904	Death	Death	15%	103%	108%
Affine	Death	Death	Death	15%	113%
HK5	Death	Death	Death	Death	800%
AD1	Death	Death	< 1%	54%	162%
3DS	Death	Death	Death	Death	900%
WWS	Death	Death	15%	800%	200%

**Fig 2:**
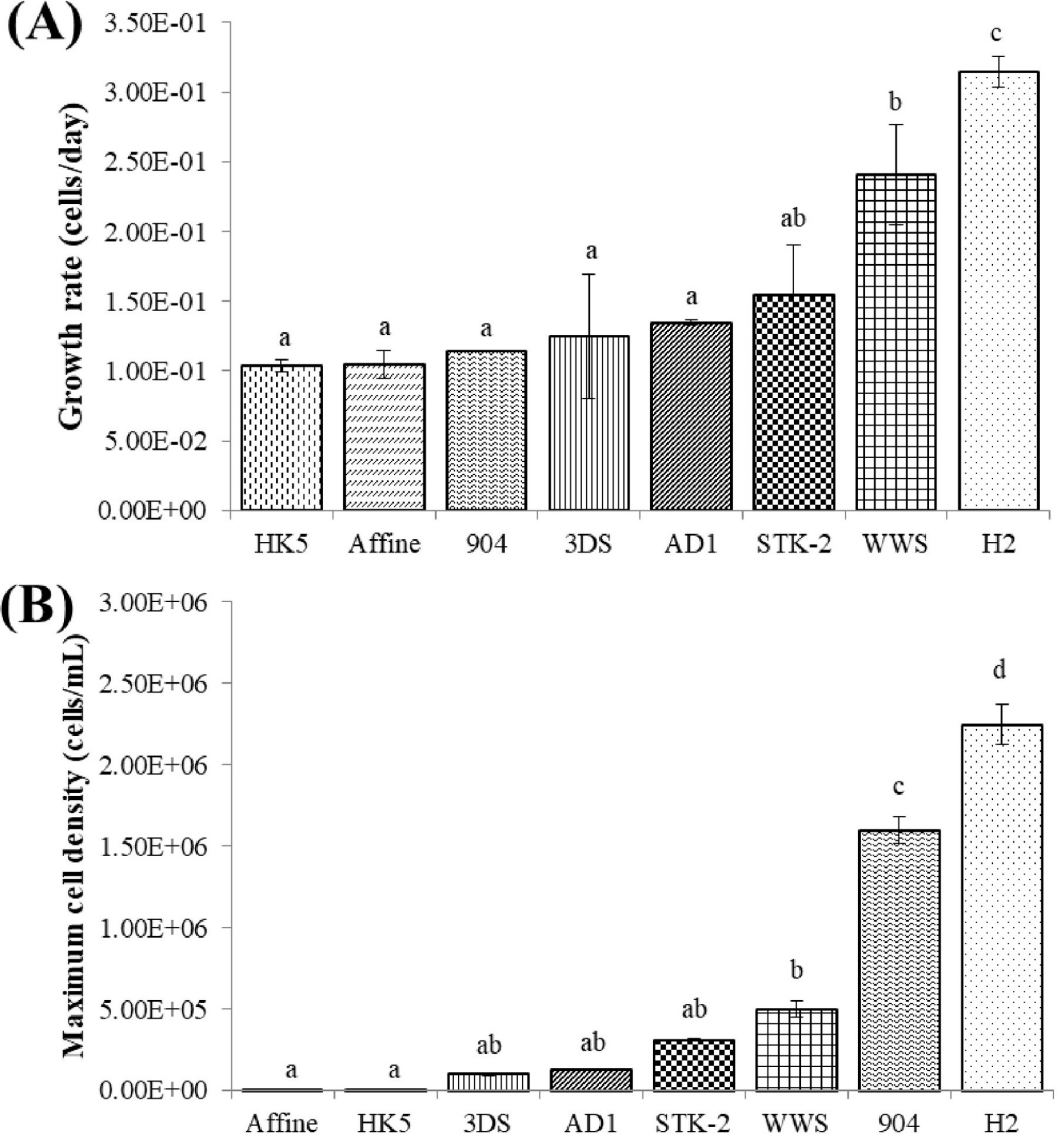
Growth rate (A) and maximum cell density (B) of eight selected algae in 10% food waste filtrate. The mean and standard deviation of three replicates are shown. Means with different letters for each treatment indicate significant differences at *p* < 0.05 according to a one-way ANOVA test.

As part of the criteria for selecting potential algal species for food waste filtrate treatment, the growth rate and maximum cell density of eight selected microalgae in 10% food waste filtrate are presented in Fig 2. The growth rate of H2 was highest among these strains, approximately 20% higher than that of the second highest strain (WWS). Excluding these two strains, the other algal species exhibited growth rates of only 0.10 to 0.16 cells d^-1^. Furthermore, H2 produced the significantly highest maximum cell density under 10% food waste filtrate treatment. The results of the growth rate and maximum cell density were consistent, suggesting that H2 is the most suitable microalga for treating food waste filtrate at a 10% concentration.

During the reproducing process, microalgae absorb nutrients such as nitrogen and phosphorus and convert light energy into chemical energy. A high growth rate of microalgae signifies high biomass production, which necessitates sufficient nutrients for algal growth. However, excessive nutrients may inhibit algal growth [30]. In this study, *C*. *aponinum* exhibited no signs of life during cultivation under 100% food waste filtrate treatment. The cell density of *C*. *aponinum* decreased from day 0 to day 2 and could not be counted in the subsequent days. Under 80% food waste filtrate treatment, the cell density of *C*. *aponinum* increased in the initial days and then followed a similar growth trend to that of the 100% food waste filtrate treatment (Fig 1). The primary reason that *C*. *aponinum* could not survive in high concentrations of food waste filtrate might be the excessive nutrients. An extremely high level of nitrogen can inhibit algal growth. For example, sufficient ammonia can provide an essential nitrogen source for microalgae through bacterial oxidation of ammonia to nitrates and nitrites in an aerobic situation, while an excessively high concentration of ammonia can be toxic to algal growth [31]. Phosphorus also plays a crucial role in microalgae growth. Similar to nitrogen requirement, an excessive level of phosphorus can negatively affect algal growth performance [13]. It has been reported that *S*. *obliquus* was significantly inhibited by excess phosphorus [32]. On the other hand, severe conditions like low pH and high total suspended solids (TSS) content might also inhibit algal growth. The source of food waste, which depends on the type of food source, is not stable [33]. Food waste filtrate might have a high TSS content, including meat granules and colloidal matter, which increases the turbidity and further affects the light transmission properties of the water body. When the light source is scattered by colloidal matter, a low photosynthesis rate would result during treatment.

### Optimum growth conditions of *C*. *aponinum*

#### Effect of light intensity on the growth of *C*. *aponinum*

**Fig 3:**
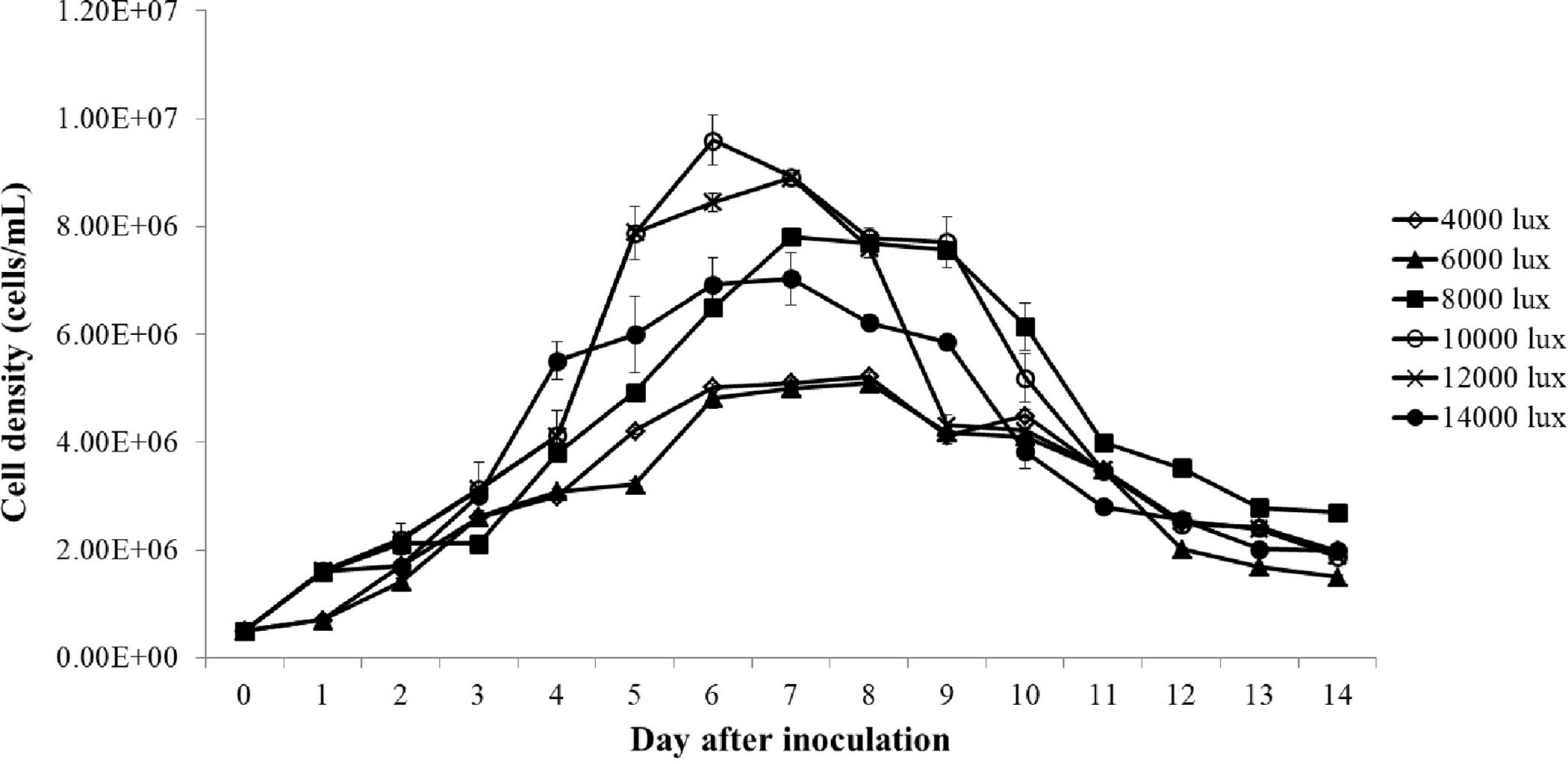
Growth of *C*. *aponinum* cultured under various light intensities. The mean and standard deviation of three replicates are shown.

**Fig 4:**
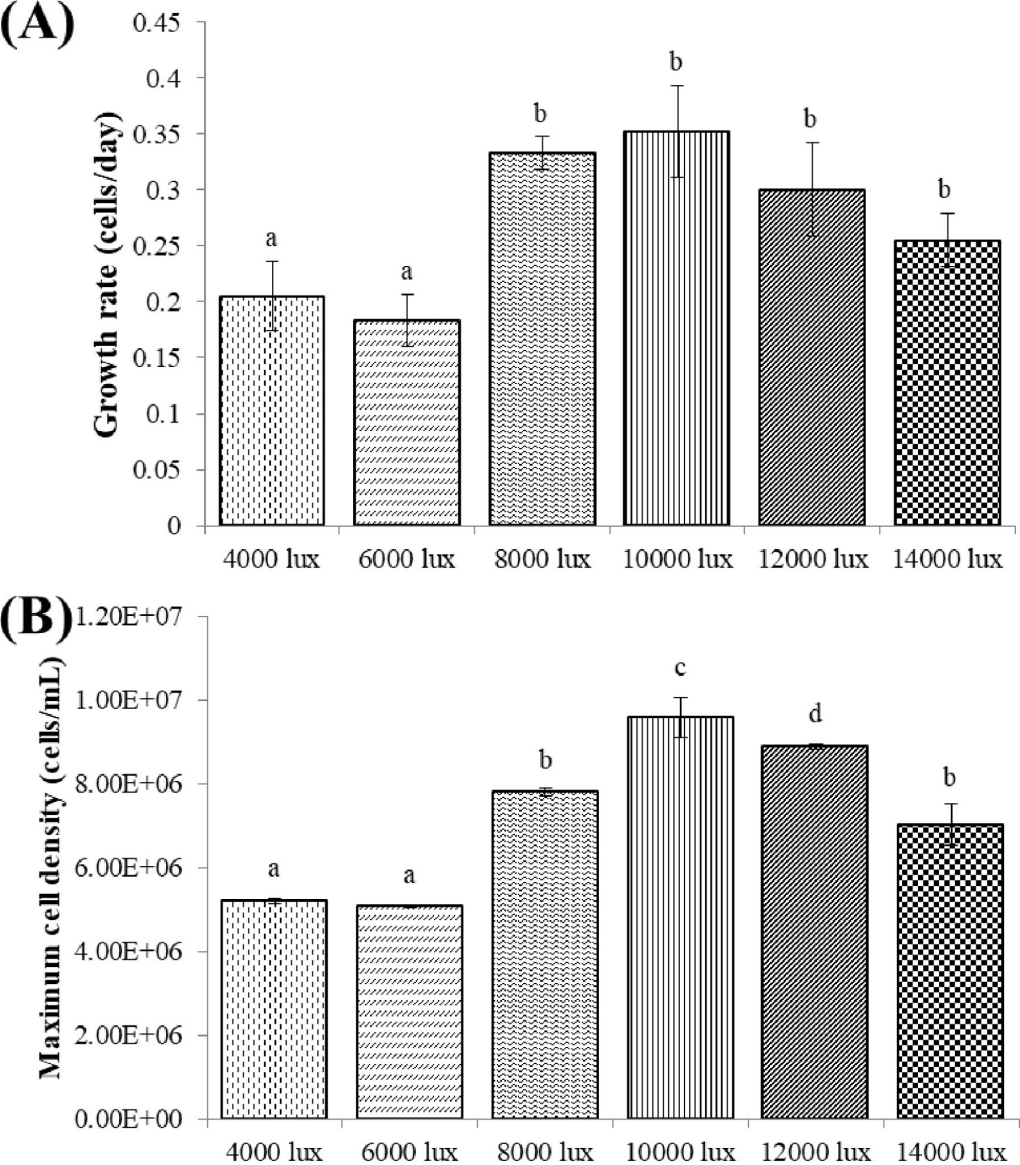
Growth rate (A) and maximum cell density (B) of *C*. *aponinum* cultured under various light intensities. The mean and standard deviation of three replicates are shown. Means with different letters for each treatment indicate significant differences at *p* < 0.05 according to one-way ANOVA test.

Fig 3 presents the growth performance of *C*. *aponinum* when cultivated under various light intensities. There was no significant difference in the growth performance of *C*. *aponinum* between different light intensities. The growth rate and maximum cell density of *C*. *aponinum* cultivated under various light intensities are shown in Fig 4. The growth rate of *C*. *aponinum* under 8,000 lux and 10,000 lux was relatively higher than the others. Additionally, the maximum cell density of *C*. *aponinum* under 10,000 lux was 9.6 × 10^6^ cells mL^-1^, which was the highest value among all the light intensity conditions. The second highest cell density was approximately 8.9 × 10^6^ cells mL^-1^ under 12,000 lux.

Light intensity is a primary factor affecting the cultivation of microalgae, significantly influencing photosynthetic kinetics and metabolite production. It is well-established that an optimal light intensity is required for the cultivation of high biomass microalgae. Both excessively high and low light intensities can inhibit microalgal growth. Low light intensity provides insufficient energy to algal photosynthetic organs, thereby limiting microalgal growth. Conversely, the algal PSII system can be easily damaged by excess light energy, resulting in reduced algal productivity [34]. Additionally, the algal photosynthetic rate may decrease due to photo-oxidation caused by exposure to high light energy [35]. In aquatic environments, photo inhibition primarily occurs under two conditions. The first is when large amounts of phytoplankton gather under serval hours of strong irradiance, a phenomenon caused by phototropism. The second occurs when algae are passively exposed to high light intensity due to physical processes, such as mixing in cultivation installations [36].

The optimal light intensity varies significantly among different microalgae species. In this study, we found that 10,000 lux was the optimal light intensity for the growth of *C*. *aponinum*, as both the growth rate and maximum cell density were highest at this level. However, this result differs greatly from those observed in other microalgal species. For example, the growth of *Anabaena flosaquae* decreased below a light intensity of 3,500 lux [37], while *Synechocystis* 6803 achieved its optimal growth rate under 3,800 lux [38]. Although a light intensity range of 3,000 to 6,000 lux is suitable for most microalgae [39], marine benthic diatoms, similar to *C*. *aponinum*, exhibited optimal growth under a light intensity of 10,000 lux [40].

#### Effect of temperature on the growth of *C*. *aponinum*

**Fig 5:**
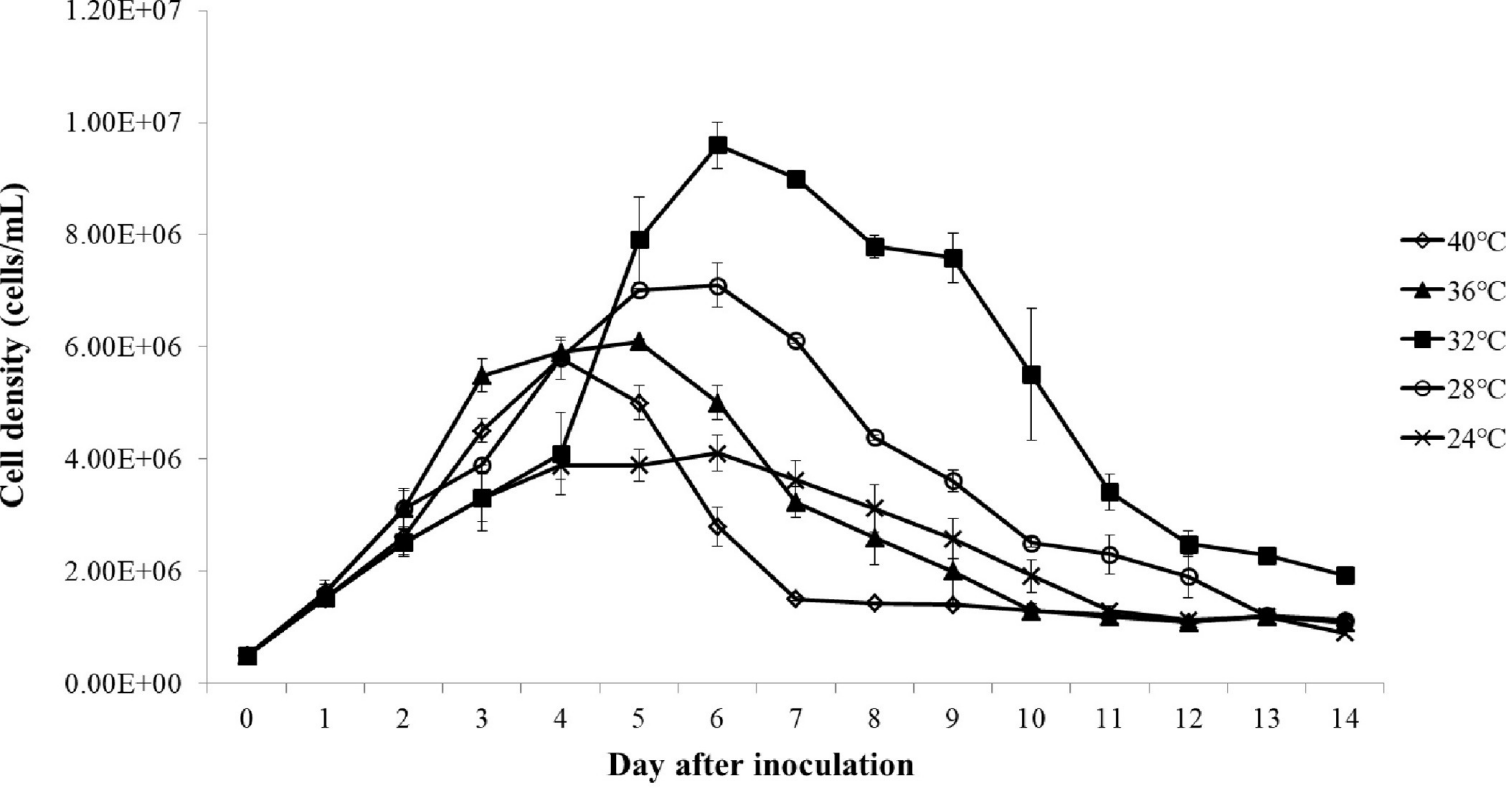
Growth of *C*. *aponinum* cultured under various temperatures. The mean and standard deviation of three replicates are shown.

**Fig 6:**
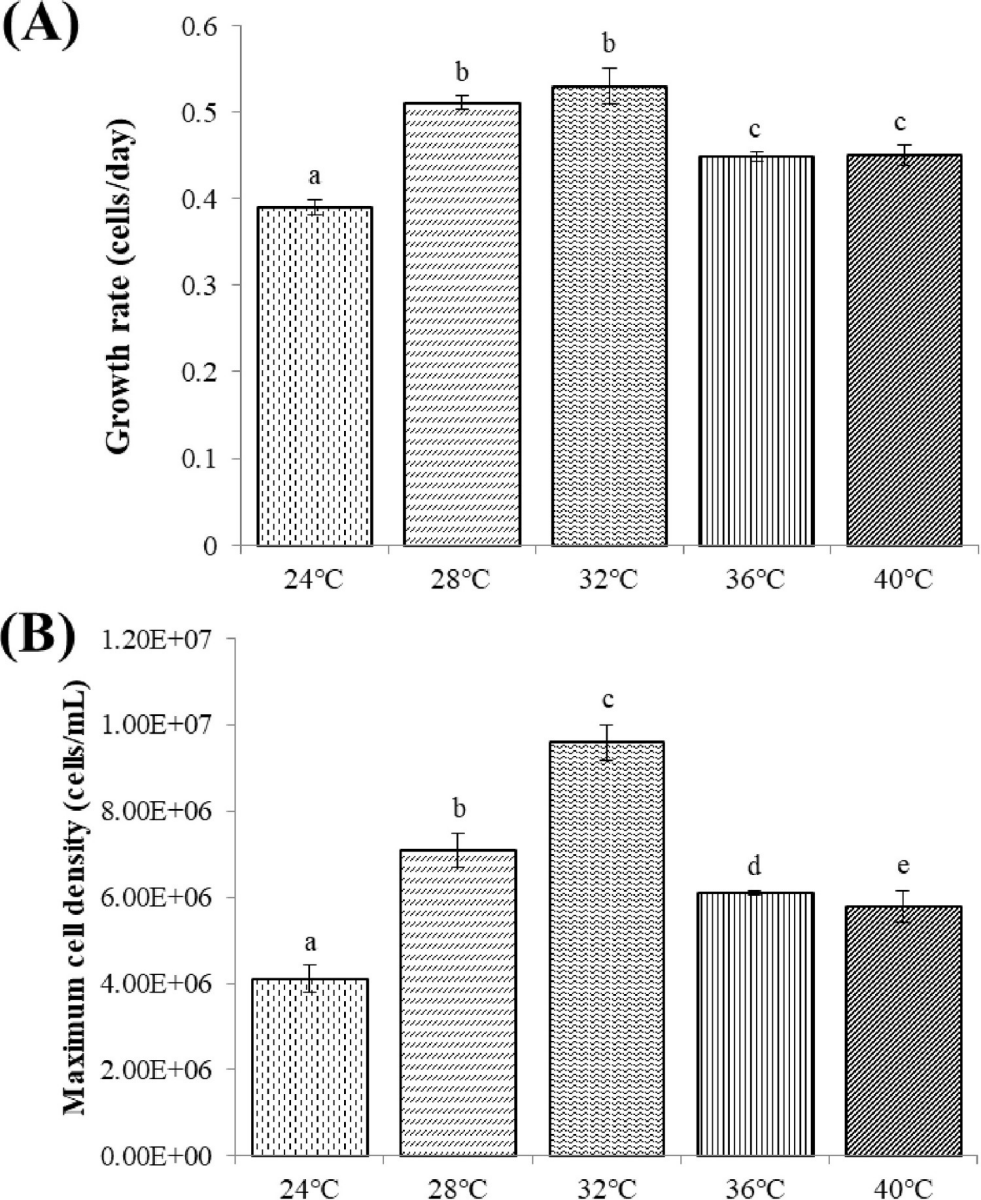
Growth rate (A) and maximum cell density (B) of *C*. *aponinum* cultured under various temperatures. The mean and standard deviation of three replicates are shown. Different letters at each treatment indicate significant differences at *p* < 0.05 according to a one-way ANOVA test.

Fig 5 presents the growth performance of *C*. *aponinum* cultivated under various temperatures. The cell density of *C*. *aponinum* increased with rising temperatures up to 32°C, but decreased with temperatures exceeding 32°C. Fig 6 illustrates the growth rate and maximum cell density of *C*. *aponinum* cultivated under various temperatures. Significant growth was observed at both 28°C and 32°C, with relatively high growth rates. The maximum cell density of *C*. *aponinum*, reaching 9.6 × 10^6^ cells mL^-1^ at 32°C, was the highest within the range of 24°C to 40°C.

The ideal temperature for algal growth facilitates the photosynthesis process without altering any biochemical or physiological conditions [41]. For mesophilic species such as *Chlorella*, the optimal temperature range is typically between 20°C and 25°C [42]. However, for thermophilic and psychrophilic species, the temperature at which maximum growth rate occurs increases to 40°C and decrease to 17°C, respectively [41]. It has been reported that *C*. *aponinum* achieved its maximum growth rate at 45°C [43], although in the present study, this was observed at 32°C. Nonetheless, not all cyanobacteria species prefer high temperatures. The four genera of *Anabaena*, *Aphanizomenon*, *Microcystis*, and *Oscillatoria* reach their maximum growth rate and cell density at approximately 25°C, and are significantly limited below 15°C [44].

Previous studies have suggested that the influence of temperature on photosynthesis may be attributed to a catalytic enzyme known as ribulose-1, 5-bisphosphate (Rubisco). In the kinetics of photosynthesis and photorespiration, Rubisco plays a role in the process as a carboxylase and oxygenase, respectively. The carboxylase activity of Rubisco has been found to increase when the temperature rises from 5°C to 50°C. However, when the temperature exceeds 30°C, the CO_2_ affinity of Rubisco decreases, leading to a reduction in biomass production due to inhibited photosynthesis, which aligns with the findings of the present study.

#### Effect of CO_2_ concentration on the growth of *C*. *aponinum*

**Fig 7:**
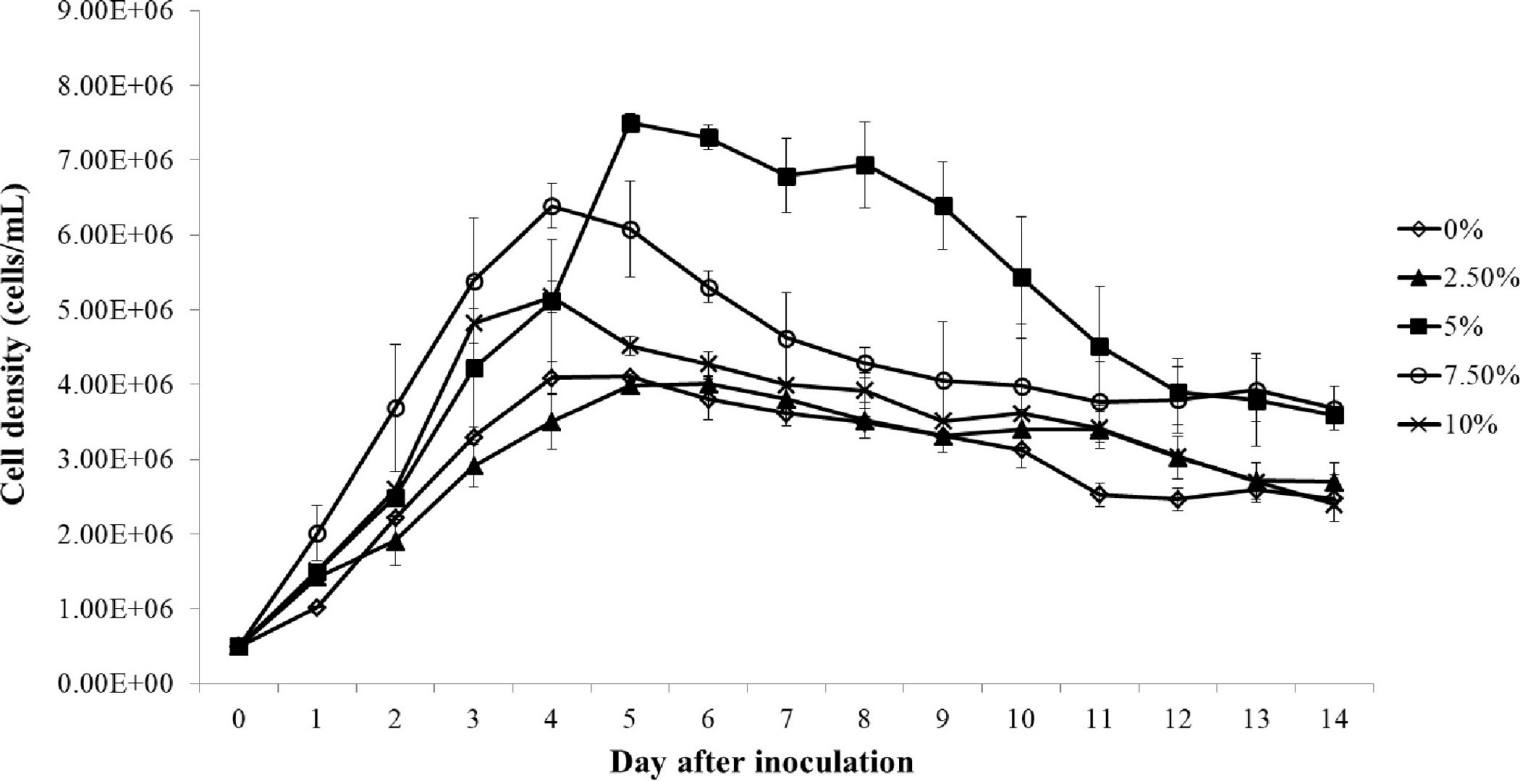
Growth of *C*. *aponinum* cultured under various CO_2_ concentrations. The mean and standard deviation of three replicates are shown.

**Fig 8:**
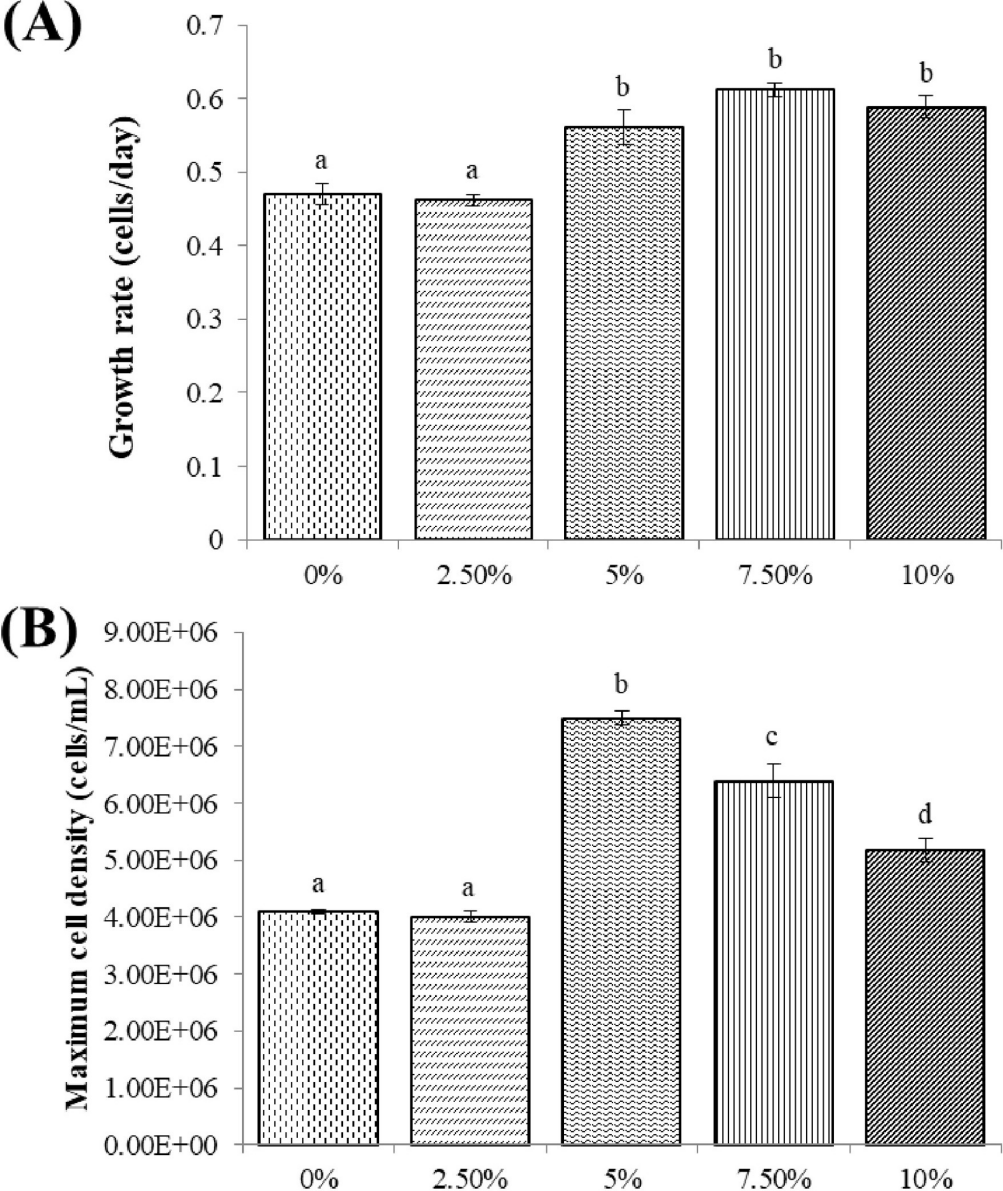
Growth rate (A) and maximum cell density (B) of *C*. *aponinum* cultured under various CO_2_ concentrations. The mean and standard deviation of three replicates are shown. Means with different letters at each treatment indicate significant differences at *p* < 0.05 according to one-way ANOVA test.

Fig 7 presents the growth performance of *C*. *aponinum* cultivated under various CO_2_ concentrations. The growth of *C*. *aponinum*, cultivated under a 5% CO_2_ concentration, was superior compared to the other conditions. The growth rate and maximum cell density of *C*. *aponinum* cultivated under various CO_2_ concentrations are depicted in Fig 8. No significant difference was observed in the growth rate of *C*. *aponinum* across different CO_2_ concentrations. However, the maximum cell density of *C*. *aponinum*, which reached approximately 7.5 × 10^6^ cells mL^-1^ under a 5% CO_2_ concentration, was significantly higher than those under other CO_2_ concentrations.

**Fig 9:**
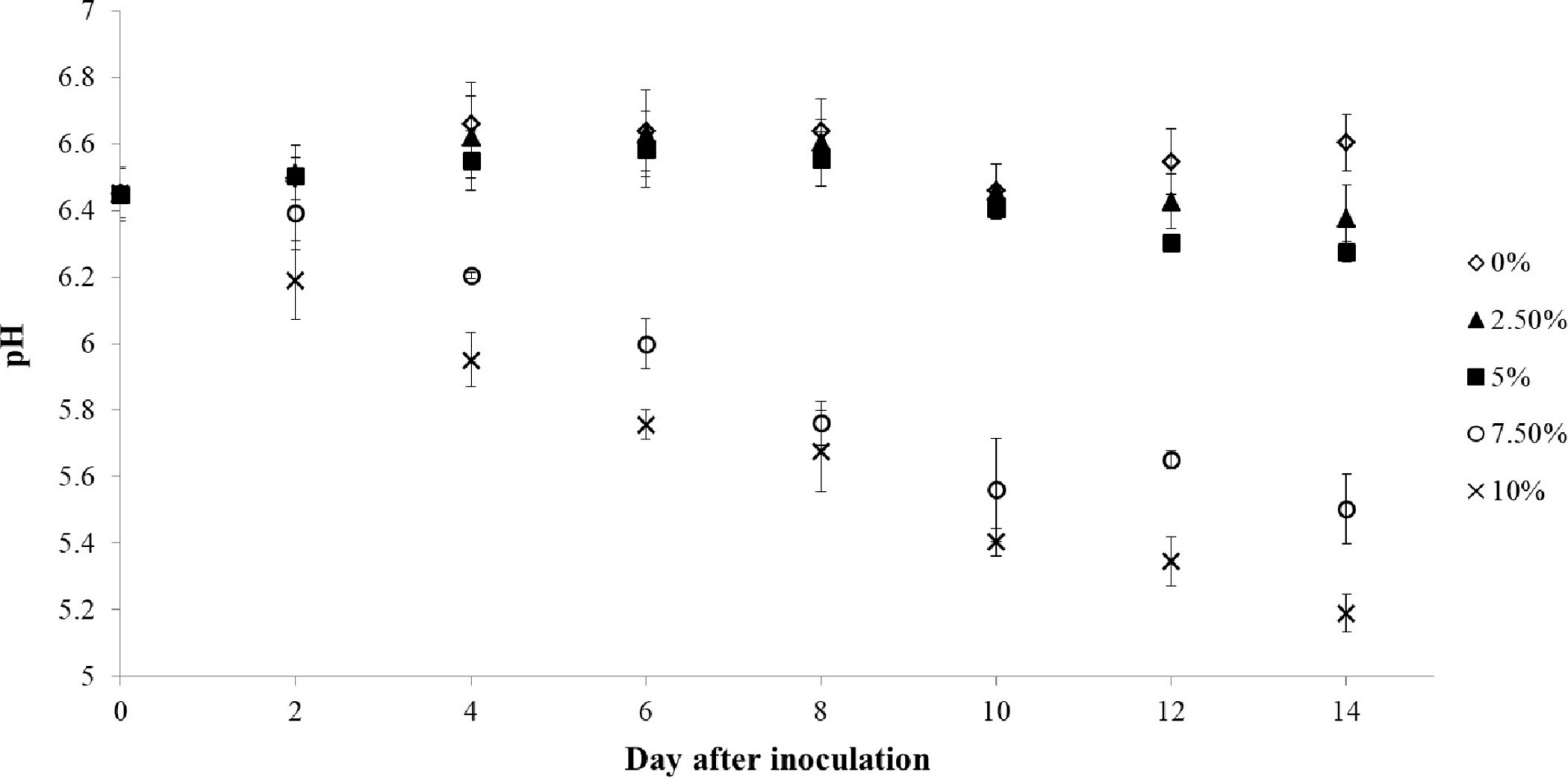
pH of the medium for cultivating *C*. *aponinum* under various CO_2_ concentrations. The mean and standard deviation of three replicates are shown.

Carbon is an essential nutrient in the algal photosynthesis process. The fixation of carbon is primarily used for respiration, which is crucial for new cell formation. The growth rate of microalgae is directly related to the rate of carbon fixation. A reduction in the carbon fixation rate equates to a decrease in the algal growth rate, as excess carbon sources can inhibit algal growth. This is due to the low absorption rate of carbon, which can acidify the survival environment. The consumption of carbon sources varies among different algal strains. However, the optimal CO_2_ concentration for microalgae cultivation is commonly 5% [45, 46], which aligns with the results for *C*. *aponinum* in the present study. As depicted in Fig 9, the pH of medium under both 7.5% and 10% CO_2_ concentrations can drop below 5.6, while it remains above 6.2 under 0%, 2.5%, and 5% CO_2_ concentrations. Therefore, it is suggested that growth inhibition under 7.5% and 10% CO_2_ concentrations may be due to acidic conditions, while the 5% CO_2_ concentration group does not exhibit the same effect.

### Efficiency of nutrient removal by *C*. *aponinum* under different CO_2_ concentrations

**Fig 10:**
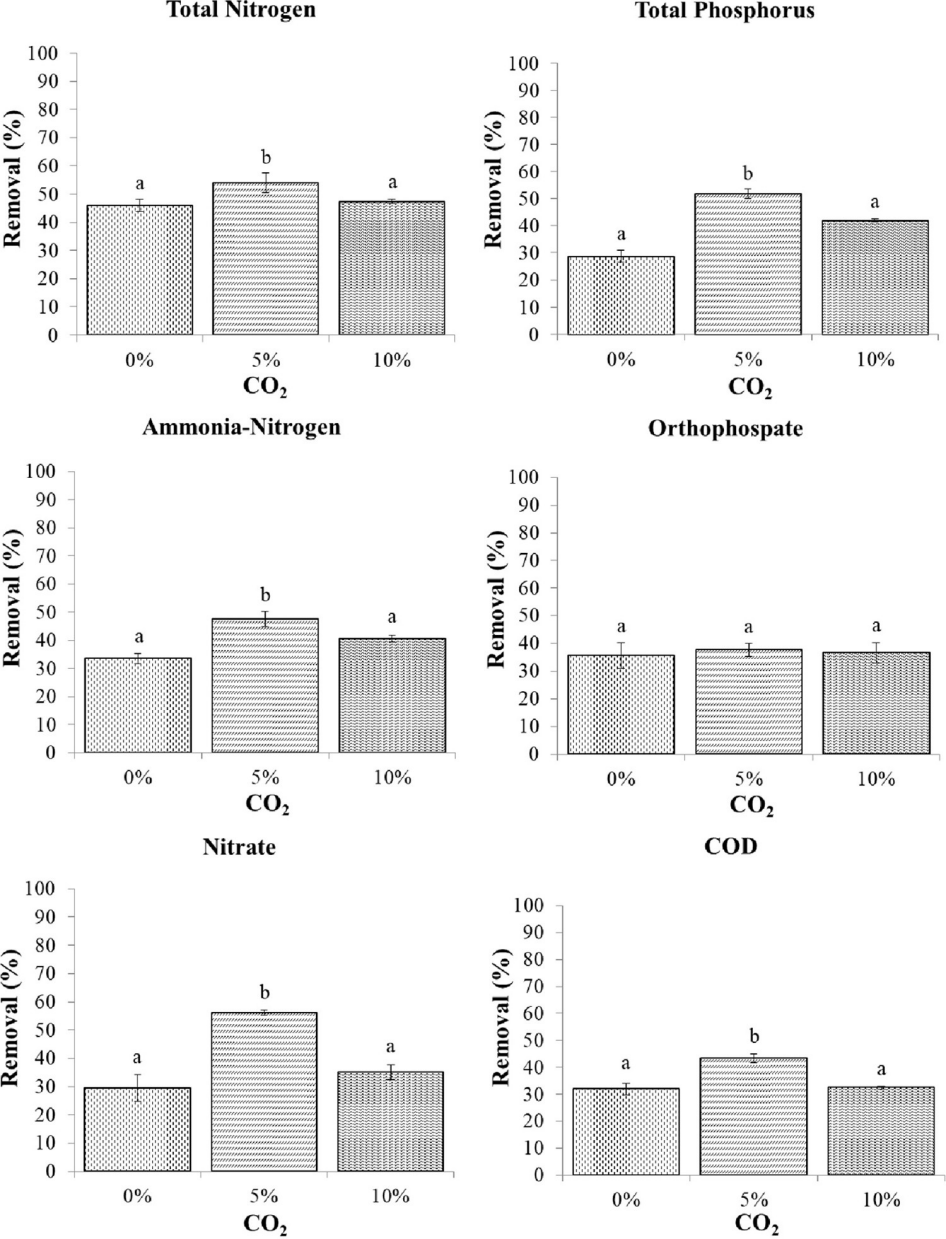
Nutrient removal efficiency of food waste filtrate treatment by *C*. *aponinum* under different CO_2_ concentrations. The mean and standard deviation of three replicates are shown. Means with different letters at each treatment indicate significant differences at *p* < 0.05 according to a one-way ANOVA test.

The variation in the removal of TN, TP, ammonia-nitrogen, orthophosphate, nitrate, and COD by *C*. *aponinum* under different CO_2_ concentrations is depicted in Fig 10 and Table 3. TN from food waste filtrate was removed by over 40% by *C*. *aponinum* under all three CO_2_ concentrations, with the removal efficiency reaching nearly 60% under a 5% CO_2_ concentration. A similar result was observed in the removal of TP, which was highest (52% removal) under a 5% CO_2_ concentration and lowest (29% removal) under a 0% CO_2_ concentration. Not only TN and TP, but also ammonia-nitrogen under a 5% CO_2_ concentration was significantly removed by *C*. *aponinum*. All of the correlated removals of orthophosphate under different CO_2_ concentrations were about 30%, which differed significantly from the results of nitrate and COD removal efficiency. For the treatment of food waste filtrate, *C*. *aponinum* significantly removed nitrate (over 50%) and COD (over 40%) under a 5% CO_2_ concentration. Collectively, our results indicate that the nutrient removal efficiency of *C*. *aponinum* was significantly high under a 5% CO_2_ concentration, except that orthophosphate was removed similarly among the three CO_2_ concentrations. Combined with the result from the previous section that *C*. *aponinum* grew well under a 5% CO_2_ concentration, it was revealed that *C*. *aponinum* had significantly increased biomass productivity and a higher efficiency to remove nutrients under a 5% CO_2_ concentration in a laboratory environment. However, further work is needed to utilize *C*. *aponinum* in food waste filtrate treatment from laboratory conditions to on-site pilot scale.

**Table 3:** The nutrient characteristics of food waste filtrate before and after treatment by *C*. *aponinum* under different CO_2_ concentrations.

	Before treatment (mg L^-1^)			After treatment (mg L^-1^)		
CO_2_ concentration	0%	5%	10%	0%	5%	10%
Total Nitrogen	20.13±0.12	20.57±0.21	20.40±0.11	10.86±0.38	9.48±0.81	10.73±0.17
Total Phosphorus	24.57±1.11	26.03±0.21	25.37±0.26	17.47±0.41	12.53±0.36	14.72±0.21
Ammonia-Nitrogen	15.71±0.30	14.78±0.39	15.66±0.18	10.46±0.32	7.73±0.19	9.30±0.29
Orthophospate	2.63±0.47	2.76±0.29	2.56±0.27	1.67±0.18	1.71±0.12	1.61±0.10
Nitrate	15.60±0.54	15.23±0.83	16.20±0.43	11.03±1.12	6.67±0.27	10.49±0.40
COD	1501.40±82.93	1491.73±29.74	1471.13±8.94	1019.78±22.67	844.91±9.24	993.93±6.68

**Fig 11:**
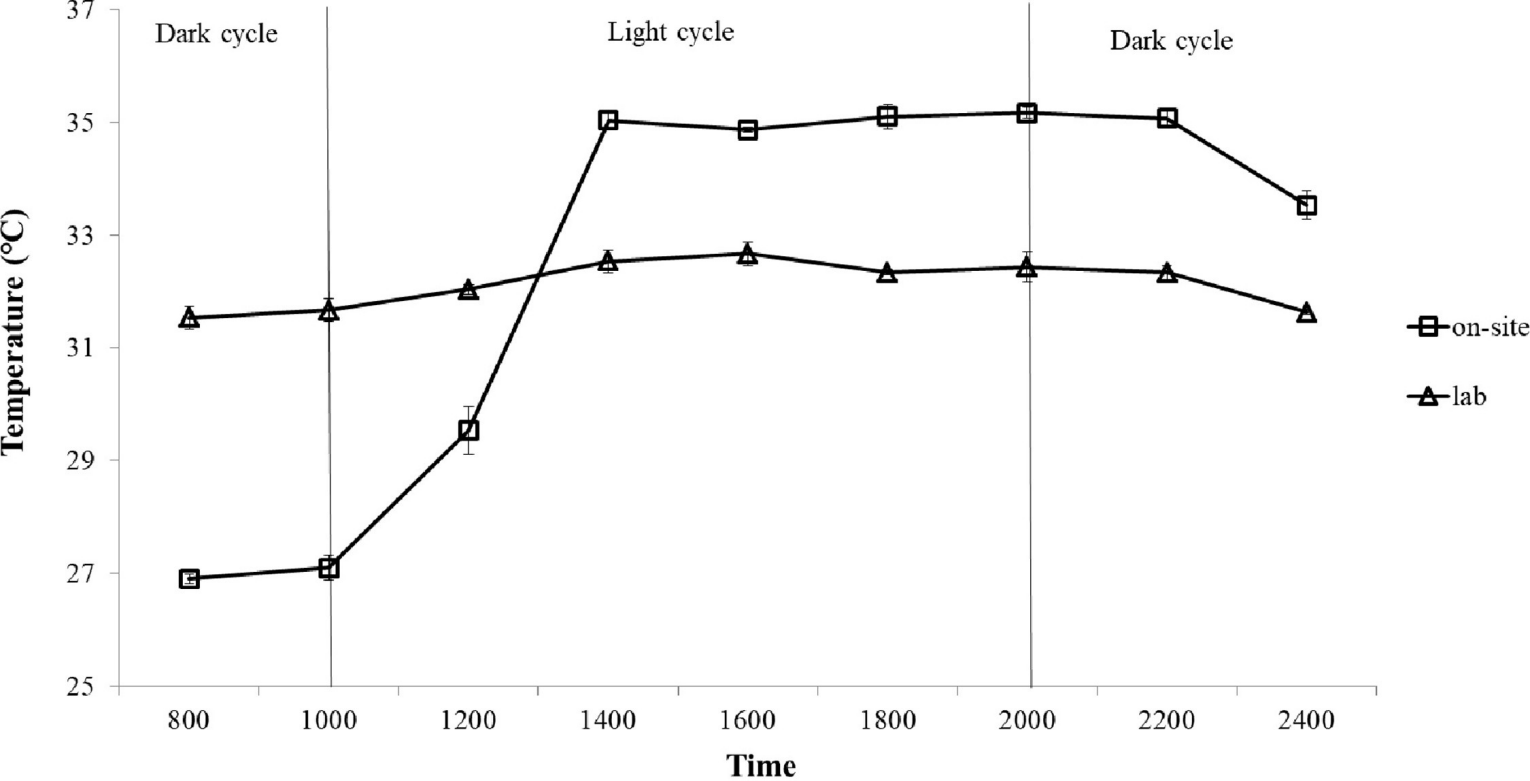
Temperature change of culture in both laboratory and on-site pilot environments. The mean and standard deviation of three replicates are shown.

**Fig 12:**
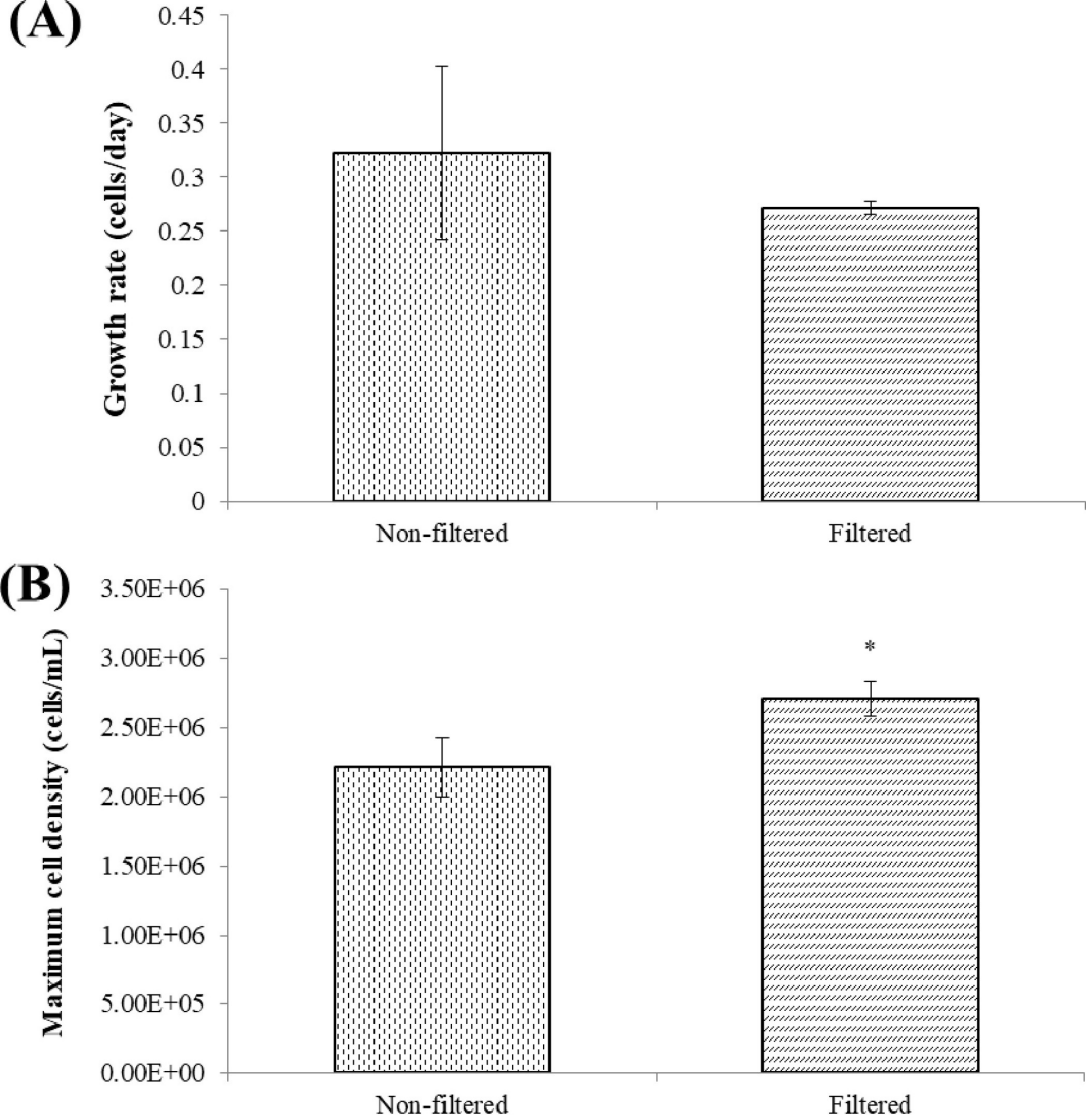
Maximum growth rate (A) and maximum cell density (B) of *C*. *aponinum* in filtered and non-filtered food waste filtrate. The mean and standard deviation of three replicates are shown. Means with different letters at each treatment indicate significant differences at *p* < 0.05 according to a one-way ANOVA test.

Comparison with the relatively stable cultivation environment in the laboratory, numerous factors affect the nutrient removal of microalgae in an on-site pilot environment. On one hand, fluctuating temperatures in the on-site pilot environment (Fig 11) might inhibit the efficiency of nutrient removal, as nutrient removal by microalgae is sensitive to temperature changes [47]. Moreover, various microalgae may exhibit different performances in nutrient removal due to their varying tolerance to temperature changes [27]. It is suggested that cyanobacteria should be applied in food waste filtrate treatment under high temperature conditions, which might be attributed to its greater adaptability to different temperatures and irradiance [47]. On the other hand, the nutrient removal efficiency might be affected by impurities or contaminations such as bacteria and fungi in the on-site pilot environment [48]. As depicted in Fig 12, non-filtered food waste filtrate might significantly influence the growth of microalgae. Furthermore, although microalgae and other microorganisms can co-exist, competition for nutrient absorption might occur [48]. However, it is challenging to filter large amounts of food waste filtrate during on-site pilot scale treatment.

### Potential application of microalgae cultivation from food waste filtrate for biofuel production

Food waste, composed of approximately 60% carbohydrates, 20% proteins, and 10% lipids [49, 50], is a valuable raw material for nutrient recovery and the production of high-value products. Unlike the conventional treatment of food waste by bacteria, this study innovatively treats food waste filtrate with microalgae. The benefits of using microalgae-derived food waste treatment include: (1) No pre-treatment is required apart from dilution. (2) In addition to providing a growth medium for microalgae, food waste filtrate offers dual potential for organic effluent treatment [51]. (3) Microalgae cultivation can facilitate the biofixation of waste CO_2_ (1 kg of dry microalgal biomass utilizes approximately 1.83 kg of CO_2_), thereby maintaining and improving air quality [52]. (4) After treating food waste filtrate, microalgae can be harvested and used for biofuel extraction [53].

Microalgae cultivation in conjunction with food waste treatment provides a pathway for the removal of organic contaminants from food waste filtrate while producing biomass for biofuel production [54]. For example, *Botryococcus braunii* has been used to remove nitrate and phosphate from sewage after primary treatment, concurrently producing hydrocarbon-rich biomass [55]. Furthermore, microalgae cultivation can yield valuable co-products such as proteins and residual biomass after oil extraction, which can be used as feed or fertilizer [33]. Anti-histamine, anti-bacterial, anti-cancer and many other valuable products can also be derived from microalgal cultures [17].

Despite its inherent potential as a biofuel resource, several challenges have hindered the development of microalgal biofuel technology to commercial viability, which would allow for sustainable production and utilization. These challenges include: (1) Species selection must balance the requirements for biofuel production and the extraction of valuable co-products [56]. (2) Higher photosynthetic efficiencies must be achieved through the continued development of production systems [57]. (3) There is a potential for a negative energy balance after accounting for requirements in water pumping, CO_2_ transfer, harvesting, and extraction [58].

## Conclusion

The current study convincingly demonstrated the feasibility of utilizing microalgae in food waste filtrate treatment under laboratory conditions. *C*. *aponinum* was identified as the most suitable microalgae for treating food waste filtrate at a 10% concentration form eight potential microalgal species cultivated in various concentrations of food waste filtrate. The optimal light intensity, temperature and CO_2_ concentration for *C*. *aponinum* cultivation were found to be 10,000 lux, 32°C, and 5%, respectively. The nutrient removal efficiency of *C*. *aponinum* was significantly high under a 5% CO_2_ concentration, ranging from 40% to 60%. However, further investigation is required to validate the potential for industrial treatment of food waste filtrate by microalgae, and considerable effort should be devoted to studying how to mitigate the variations that occur in on-site pilot scale cultivation.

## Supporting information

S1 Data set (XLSX)
